# What Does Food Retail Research Tell Us About the Implications of Coronavirus (COVID-19) for Grocery Purchasing Habits?

**DOI:** 10.3389/fpsyg.2020.01448

**Published:** 2020-06-05

**Authors:** Rosemarie Martin-Neuninger, Matthew B. Ruby

**Affiliations:** ^1^MacMillan Brown Centre for Pacific Studies, University of Canterbury, Christchurch, New Zealand; ^2^Department of Psychology and Counselling, La Trobe University, Wodonga, VIC, Australia

**Keywords:** COVID-19, grocery shopping, food choice, heuristics, panic buying

## Introduction

The global pandemic of COVID-19 (a disease caused by a novel coronavirus, SARS-CoV-2) has caused drastic changes in the structure of people's daily routines in many countries around the world, including the way we purchase food. Many regions and countries have “locked down” to prevent the spread of the virus, including first Wuhan in China, and countries such as the USA, UK, Germany, India, Poland, and New Zealand. During these lockdown periods, people are only allowed to leave their homes if strictly necessary—e.g., for essential work, exercise, medical care, or to purchase groceries or supplies. Although these lockdowns were initially declared to last for a matter of weeks, Ferguson et al. (2020) suggest that lockdowns may need to be implemented regularly for over a year, in order to manage the burden of COVID-19 on health services.

This opinion paper evaluates the emerging and likely consequences of these lockdowns on consumer grocery purchasing habits and their implications for food retail as of early April of 2020, with a particular focus on New Zealand as a case study. In doing so, we draw on classic (e.g., Nowlis, 1995; Suri and Monroe, 2003) and recent (e.g., Sproesser et al., 2018; Bialkova et al., 2020) research on consumer behavior, and identify key areas in need of further research.

## Consumer Behavior Prior To Lockdown

Prior to the lockdown, many governments and food suppliers assured shoppers that food supply chains to supermarkets were fully functioning (FGC, 2020; Newshub, 2020). However, in the weeks before the lockdown was announced, many individuals began to panic buy and stock-pile products such as water, gloves, carbohydrate-rich staples (e.g., bread, pasta), canned food, hand sanitizer, and even toilet paper (Mao, 2020).

Panic buying is a common response at times of fear and uncertainty, and can be seen as rational (e.g., stockpiling essential goods that are in limited supply) or irrational (e.g., stockpiling non-essential products that are not in limited supply; Sterman and Dogan, 2015). Even though supply chains were operating as normal, the panic buying itself caused shortages of many products on the supermarket shelves. Given that people are especially influenced by the behavior of their peers at times of uncertainty (Sherif, 1936; Pickett and Gardner, 2005; Smith et al., 2007), seeing others engage in panic buying likely exacerbated the situation. The recent cases of panic buying were not novel—during the SARS epidemic, misinformation and rumors spread via the internet and SMS communications were also linked to waves of panic buying (e.g., Ding, 2009).

## Consumers' Shifting Grocery Shopping Experience

Following the outbreak of a global pandemic of COVID-19, the retail experience has dramatically changed for the foreseeable future. In many countries, the only retail outlets open are supermarkets, and the retail experience is now much different. Supermarkets in New Zealand, Europe, Australia, the USA, and the UK have introduced changes to reduce panic buying and to minimize the spread of COVID-19 while shopping, although these changes vary considerably from store to store. Some of the more common measures are described below.

Supermarkets have placed a temporary limit for their customers of buying two or three similar items per shopping visit, reduced their opening hours, and asked people to “shop normal” (Coles, 2020; Countdown, 2020a; nzherald.co.nz, 2020), in order to mitigate the effects of panic buying.

To minimize the spread of the virus, shoppers are required to maintain a distance from other customers and staff of 1.5–2 m (Australian Government Department of Health, 2020; New Zealand Government, 2020). Check-out staff are protected by Perspex screens and protective visors or masks (The News, 2020). Customers have been asked to shop alone, and once stores have reached the maximum safe capacity, people are only allowed to enter the supermarket on a “one in, one out” basis, with floor-markings at checkouts directing customers to maintain their distance (New World, 2020; Waitrose, 2020). In some supermarkets, customers have to follow floor signage through the supermarket and cannot go backwards if they forget something (Tesco, 2020). Other supermarkets have closed deli counters and salad bars to avoid close contact with their customers (World Economic Forum, 2020).

Like other viral diseases, there is the possibility that food shoppers might contract COVID-19 by simply touching a surface or object that has the virus on it (CDC, 2020). Supermarkets are now providing wipes for cleaning trolley/basket handles and hand sanitizing units, which are placed around the store (New World, 2020; Tesco, 2020).

To buy groceries, consumers usually touch cash or credit cards. Supermarkets are advising their customers to pay in a cashless or contactless way (e.g., using payWave or Tap & Go) to minimize the handling of cash (New World, 2020; Waitrose, 2020) and reduce the chance of the virus spreading through this means (Seymour et al., 2020). Customers have now to pack their own bags (New World, 2020) and fewer checkouts are operating so that customers maintain physical distance from staff and one another (Waitrose, 2020).

## Implications of Constraints on The Shopping Process

As a result of the many changes to the shopping process, consumers now have fewer opportunities to interact with retail staff, and may feel pressure to shop quickly, to minimize time in contact with others in the supermarket. It is then likely that many choices will be made before consumers enter the supermarket, such that any lengthy decision-making processes happen away from the other customers and staff. This has implications for retailers and marketers. Previous research has shown that consumers' shopping habits vary depending on time constraints and motivation to search for information (Iyer, 1989; Park et al., 1989; Liu et al., 2017), so it is inevitable that the products that people purchase will change as a result of the recent changes.

### Use of Price and Brand Heuristics

The majority of consumers go to the supermarket with a specific goal in mind and frequently bring a shopping list (Bialkova et al., 2020). Under time constraints, most people will not have time to process product information, and rely more heavily on heuristics such as brand name, price, product images, and color-coded labels to make their food choices (Nowlis, 1995; Schulte-Mecklenbeck et al., 2013; Bialkova et al., 2020). When consumers have more time available, they tend to more carefully consider information presented on the product label (Silayoi and Speece, 2004), and engage in less impulse buying (Hausman, 2000). Suri and Monroe (2003) show that motivation to find information also plays a role, such that when under time pressure, people motivated to process information rely on these heuristics more than those who are not motivated. Under the current shopping conditions, the use of these heuristics is likely to increase. As a result, consumers under time constraints are predicted to choose high price and high-quality brands over low-quality and low-price brands, and products with enhanced features (Bialkova et al., 2020) over more basic products.

### Demand for Online Shopping

Since the lockdown, supermarkets have created systems to more safely deliver groceries—e.g., in New Zealand, New World Supermarket introduced contactless deliveries for people who ordered online (New World, 2020). However, demand for online shopping has dramatically increased, similar to what happened during the SARS epidemic (e.g., Kee and Wan, 2004; Forster and Ya, 2005), and this demand has exceeded the capacity of supermarkets in many countries. As such, many supermarkets are restricting this service to those particularly in need (e.g., people over 70 years of age, people with chronic illness, people who are in self-isolation, people with disabilities; Countdown, 2020a). It is likely that due to these restrictions, many consumers will not have a positive experience and will not be satisfied with the service. No New Zealand supermarket is currently offering a ‘live customer support chat' to provide customers with immediate help with accessing their services. However, to cope with this increased demand, many retail shops are expanding their online capabilities—e.g., Countdown has just launched the first national e-store supermarket (Countdown, 2020b).

### Grocery Expenditures

During the Great Recession of 2008, consumer spending on supermarket foods increased (Cha et al., 2015), while spending on many other product categories decreased. This trend is likely to be visible in the expected recession due to COVID-19, as opportunities to spend at other outlets are greatly restricted. Disruptions in the supply change may cause supply limitations and a further increase in prices. However, supermarket spending will likely vary depending on the income (support) that consumers have available during and after the pandemic.

## Discussion

Generally, people purchase according to food habits and liking (Sproesser et al., 2018). Under the new shopping conditions of the COVID-19 pandemic, food choices will depend heavily on product availability and restrictions on how many products consumers can buy. We can expect that consumers will predominantly use simple heuristics such as brand and price, particularly with products for which they usually spend time looking at labels and packaging at the point of sale. This might have the effect that consumers spend more overall per item than they did previously, raising their overall supermarket spend and decreasing spending in other areas. Prices may also rise due to restricted supply. Although people will continue to make purchases in line with their individual taste preferences, habits, and health beliefs, given the present restrictions on the availability of some food products, they may need to sometimes buy unfamiliar brands. Given that many consumers will be spending less time in-store than usual, key brands and products will need to be immediately visible to consumers, and be marketed outside of the point of sale environment—e.g., on TV and internet advertisements. Easily-recognized, well-established brands (where available) should therefore have an advantage over newer and less prominent brands.

To be competitive, food companies will need to deliver a customer-centric experience that make it enjoyable for people to go to the supermarket, without compromising staff or customer safety. Supermarkets will also need to fine-tune their online delivery services to maintain consumers' trust and confidence in the face of elevated demand. Researchers and retailers should investigate specific ways in which consumers changed their grocery shopping behavior during the lockdown (e.g., frequency of shopping trips, time spent in store, use of heuristics vs. deliberate product comparisons, purchase of ingredients vs. ready-made meals) and to what extent they plan to continue shopping in this way.

## Author Contributions

RM-N created the first draft of the manuscript and both authors contributed to all further drafts.

## Conflict of Interest

The authors declare that the research was conducted in the absence of any commercial or financial relationships that could be construed as a potential conflict of interest.
